# Hepatitis-related adverse events associated with immune checkpoint inhibitors in cancer patients: an observational, retrospective, pharmacovigilance study using the FAERS database

**DOI:** 10.3389/fphar.2024.1383212

**Published:** 2024-06-14

**Authors:** Zhiwen Fu, Jinmei Liu, Cong Zhang, Huiping Hu, Shijun Li, Yu Zhang, Ruxu You

**Affiliations:** Department of Pharmacy, Union Hospital, Tongji Medical College, Huazhong University of Science and Technology, Wuhan, China

**Keywords:** immune checkpoint inhibitors, hepatitis, disproportionality analysis, pharmacovigilance study, FAERS database

## Abstract

**Background:** Immune checkpoint inhibitors (ICIs), including anti-PD-1, anti-PD-L1 and anti-CTLA-4 antibodies, have become a standard treatment for multiple cancer types. However, ICIs can induce immune-related adverse events, with hepatitis-related adverse events (HRAEs) being of particular concern. Our objective is to identify and characterize HRAEs that exhibit a significant association with ICIs using real-world data.

**Methods:** In this observational and retrospective pharmacovigilance study, we extracted real-world adverse events reports from the FDA Adverse Event Reporting System database spanning from the first quarter of 2004 to the first quarter of 2023. We conducted both Frequentist and Bayesian methodologies in the framework of disproportionality analysis, which included the reporting odds ratios (ROR) and information components (IC) to explore the intricate relationship between ICIs and HRAEs.

**Results:** Through disproportionality analysis, we identified three categories of HRAEs as being significantly related with ICIs, including autoimmune hepatitis (634 cases, ROR 19.34 [95% CI 17.80–21.02]; IC025 2.43), immune-mediated hepatitis (546 cases, ROR 217.24 [189.95–248.45]; IC025 4.75), and hepatitis fulminant (80 cases, ROR 4.56 [3.65–5.70]; IC025 0.49). The median age of patients who report ICI-related HRAEs was 63 years (interquartile range [IQR] 53.8–72), with a fatal outcome observed in 24.9% (313/1,260) of these reports. Cases pertaining to skin cancer, lung cancer, and kidney cancer constituted the majority of these occurrences. Patients treated with anti-PD-1 or anti-PD-L1 antibodies exhibited a higher frequency of immune-mediated hepatitis in comparison to those undergoing anti-CTLA-4 monotherapy, with a ROR of 3.59 (95% CI 1.78–6.18). Moreover, the dual ICI therapy demonstrated higher reporting rates of ICI-related HRAEs compared to ICI monotherapy.

**Conclusion:** Our findings confirm that ICI treatment carries a significant risk of severe HRAEs, in particular autoimmune hepatitis, immune-mediated hepatitis, and hepatitis fulminant. Healthcare providers should exercise heightened vigilance regarding these risks when managing patients receiving ICIs.

## 1 Introduction

Since the elucidation of the role of immunological processes in tumorigenesis, multiple immune checkpoint inhibitors (ICIs) targeting immune checkpoint molecules have emerged as promising cancer immunotherapies (Galluzzi et al., 2020; Robert, 2020). These include inhibitors of cytotoxic T lymphocyte-associated protein 4 (CTLA-4), programmed cell death 1 (PD-1), and programmed death ligand 1 (PD-L1) (de Miguel and Calvo, 2020; Bagchi et al., 2021). By blocking these immune checkpoint proteins, ICIs can enhance T cell-mediated anti-tumor immunity. Since ipilimumab, as the first CTLA-4 inhibitor, was approved by the US Food and Drug Administration (FDA) for advanced melanoma in 2011, the ICIs have revolutionized the treatment landscape across various malignancies and have become an intensely studied area of cancer research (Dall'Olio et al., 2022; Vafaei et al., 2022).

However, the expanding clinical utilization of ICI agents has revealed a broad range of immune-related adverse events (irAEs) (Martins et al., 2019). It has been demonstrated that irAEs are caused by excessive immune activation affecting multiple organs, particularly the skin, liver, endocrine system, and gastrointestinal tract (Thapa et al., 2019; Albandar et al., 2021). As a key site of drug metabolism, the liver is a frequently impacted organ during cancer immunotherapy and the hepatotoxicity resulting from ICIs treatment is typically classified as immune-mediated hepatitis (Ng et al., 2022). Hepatitis has been reported as the third most common toxicity (5%–10%) following the dermatologic (44%–68%) and gastrointestinal (35%–50%) irAEs (Tian et al., 2018; Wang et al., 2020). Hepatitis, which is an inflammation of the liver, can be induced by ICIs due to their impact on immune system tolerance and regulation. The occurrence of hepatitis in patients treated with ICIs ranges from mild elevations in liver enzymes to severe hepatotoxicity. This severe form can lead to significant risks, including the development of liver cancer in chronic cases. Several mechanisms were suggested for the association between ICI therapies and the development of hepatitis. One potential mechanism of ICI-induced liver toxicity is the direct effect on liver cells. The presence of PD-1 and PD-L1 on the normal tissues cells implies that the use of ICIs could activate the body’s complement system against these non-cancerous “self” cells (Parlati et al., 2023). Another possible mechanism is the disturbance of immune homeostasis, characterized by the expansion of proinflammatory T helper cell subsets (Th1, Th17) and subsequent release of cytokine release (IL-2, IFN-γ, TNF-α) (Adams et al., 2010; Liu et al., 2023). In addition, ICI-mediated monocyte activation and inflammatory milieu generation may also contribute to immune-mediated hepatitis (Shojaie et al., 2021).

The pharmacovigilance studies on irAEs associated with ICIs treatment have identified several possible clinical toxicities to help guide medical practice and enhance patient care, as well as the hepatotoxicity with different ICIs (Salem et al., 2018; Gérard et al., 2021; Ma et al., 2021; Xu et al., 2023). The previous work identified various liver-related adverse events reported with different ICIs (Xu et al., 2023), but the studies focusing on association between ICI therapy and hepatitis-related adverse events (HRAEs) remain limited. Herein, in this observational, retrospective, pharmacovigilance study, we aim to utilize a disproportionality analysis, based on real-world adverse events reports from the FDA Adverse Event Reporting System (FAERS) database, to conduct a comprehensive assessment of HRAEs associated with ICIs and to provide a detailed description of the clinical features of reported cases pertaining to ICI-related HRAEs. The findings from this study will provide a valuable reference for healthcare providers to caution the risk of HRAEs when managing patients receiving ICIs.

## 2 Methods

### 2.1 Study design and data sources

This retrospective, observational pharmacovigilance study utilized disproportionality analysis of adverse drug reaction reports from the FAERS database. The FAERS database is a comprehensive, publicly accessible passive surveillance system incorporating global data on medication-related adverse events and errors submitted by healthcare professionals, patients, and pharmaceutical companies in the United States and worldwide (Ma et al., 2021; Feng et al., 2022). Our study encompassed all adverse event reports in FAERS ranging from the first quarter of 2004 (Q1 2004) to the first quarter of 2023 (Q1 2023). Relevant adverse event data was obtained using the immune checkpoint inhibitors, including CTLA4 inhibitors (ipilimumab and tremelimumab), PD-1 inhibitors (nivolumab, pembrolizumab, and cemiplimab), and PD-L1 inhibitors (atezolizumab, avelumab, and durvalumab), as the primary suspected (PS) drugs. Since the ICI-based combination therapies are usually used in the clinical settings, the dual ICI therapy (CTLA4 inhibitor and PD-1/PD-L1 inhibitors) and ICI combined with chemotherapy were also included in the analysis for the investigation of differences. All adverse events in FAERS are coded using the preferred terms (PTs) based on the Medical Dictionary for Regulatory Activities (MedDRA version 26.1).

### 2.2 Data processing procedure

We exacted all quarterly data extract (QDE) data from the FAERS ranging from 2004Q1 to 2023Q1, which is available at: https://fis.fda.gov/extensions/FPD-QDE-FAERS/FPD-QDE-FAERS.html. The files listed on this page contain raw data extracted from the FAERS database for the indicated time and we can choose the desired quarter to download for analysis. The QDE file contains diverse data, including demographics and administrative details (DEMO), drug information (DRUG), adverse events (REAC), patient outcomes (OUTC), reporting sources (RPSR), treatment timelines (THER), and dosage indications (INDI). All FAERS data is recorded in either ASCII or XML formats, the ASCII files were used as the data sources and imported into SAS software (version 9.4). To ensure data integrity and preclude duplication, a deduplication process recommended by the FDA was implemented based on two criteria: i. When the unique case identifier (CASEID) was identical, the most recent FDA receipt date (FDA_DT) was selected; ii. For reports with identical CASEID and FDA_DT, the higher PRIMARYID number (the unique identifier assigned to each report) was chosen (Giunchi et al., 2023).

**FIGURE 1:**
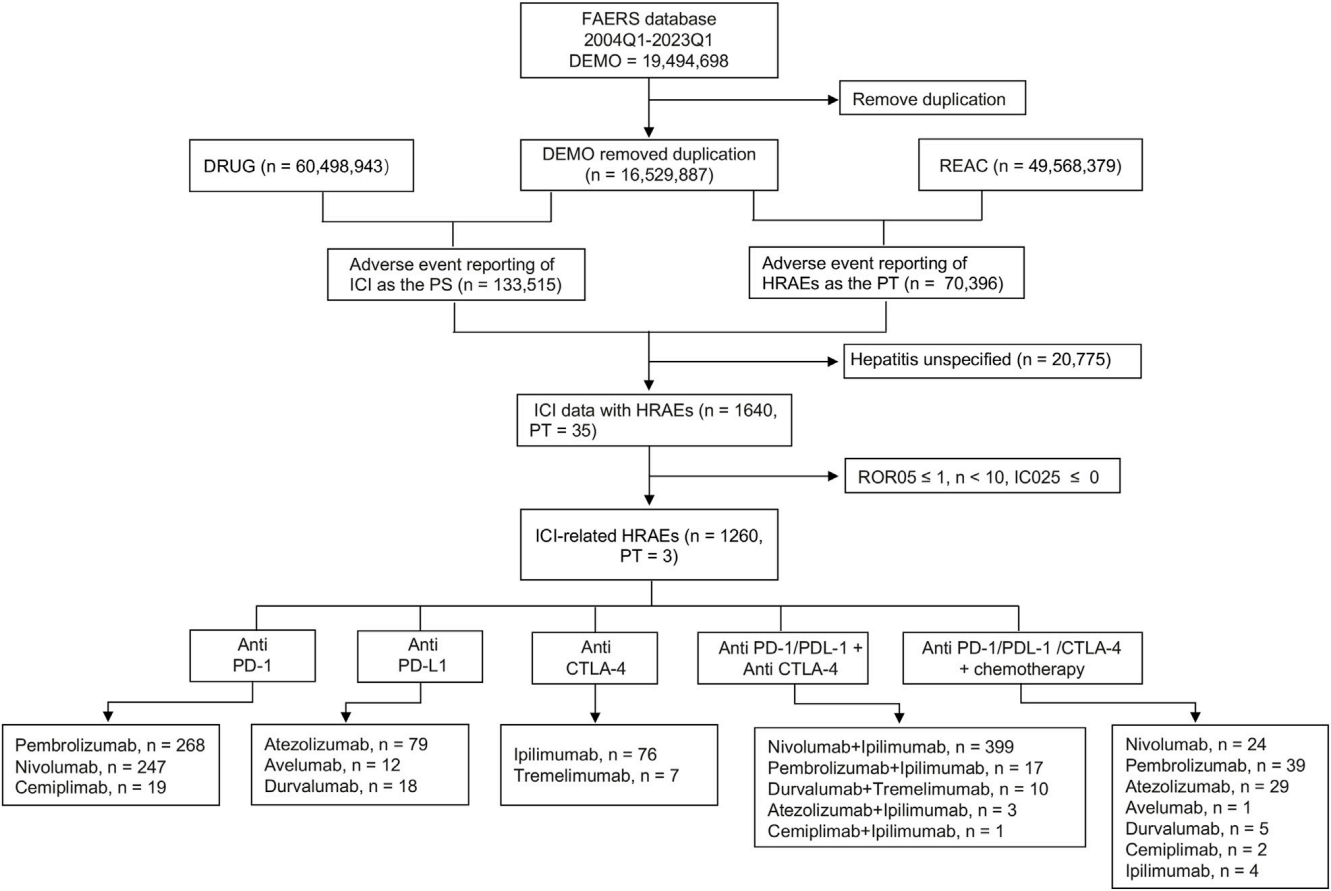
Flow chart showing the selection process of hepatitis-related adverse events for immune checkpoint inhibitors (ICIs) in the Food and Drug Administration Adverse Event Reporting System (FAERS). HRAEs, hepatitis-related adverse events; PTs, preferred terms; PS, primary suspected drugs; ROR05, the lower limit of 95% CI of reporting odds ratio (ROR); IC025, the lower limit of 95% CI of the information component (IC).

Our initial inquiry focused on the occurrence of HRAEs in patients subjected to ICIs, as documented in the FAERS database. The comprehensive data processing methodology is demonstrated in Figure 1. Starting with a dataset containing 19,494,698 adverse event reports, we conducted a thorough deduplication process using the PRIMARYID and/or CASEID recorded in the DEMO files, ultimately obtaining 16,529,887 unique adverse event reports for analysis. Among these, there were 133,515 adverse event reports associated with the use of various ICI drugs and a cumulative total of 70,396 hepatitis adverse events were cataloged as PTs in the FAERS database. To ensure specificity in our analysis, cases involving non-specific HRAEs were excluded. Consequently, we obtained a refined dataset of the ICI drugs reporting HRAEs (1,640 cases, 35 PTs).

### 2.3 Signal mining

Disproportionality analysis was utilized in this study to evaluate reporting patterns of suspected ICI-related hepatitis adverse events compared to other drugs in the FAERS database. In order to improve the rigorousness of our analysis, both the Bayesian method and the frequency method were simultaneously applied in our study. Frequency methods demonstrate greater sensitivity compared to Bayesian analyses, whereas Bayesian methods exhibit higher specificity. (Shen et al., 2019). In the present study, disproportionality was quantified by the information component (IC, a method originally introduced through the Bayesian Confidence Propagation Neural Network) and reporting odds ratio (ROR) (Shu et al., 2023; Trillenberg et al., 2023). The IC method can provide a conservative correlation measure and reduce the risk of highlighting spurious associations, especially for events with very low expected frequencies in large databases (Hou et al., 2014). The ROR allows to estimate the relative risk and identify abnormally higher than expected proportions of adverse event reporting, hence highlighting the risks associated with the use of specific drugs (Rothman et al., 2004). Specific formulas for calculating the IC and ROR along with their 95% confidence interval (CI) are shown below:

$$IC={\mathrm{Log}}_{2}\left(\frac{N_{observed}}{N_{expected}}\right)$$

$$N_{expected}=\frac{N_{drug}*N_{event}}{N_{total}}$$

where N_expected_ is the number of hepatitis records expected for the ICI. N_observed_ is the number of hepatitis records for the ICI. N_drug_ is the number of all adverse event reports associated with ICI agents. N_event_ is the number of hepatitis adverse events reported in the full database. N_total_ is the number of all adverse event reports for all drugs in the full database. The IC025 represents the lower boundary of the 95% credibility interval for the IC, which serves as a statistical measure. Traditionally, a positive value exceeding zero is considered the threshold for detecting signals. In our analysis, we also estimated the disproportionality of hepatitis adverse events among different ICI treatment strategies using the ROR along with its corresponding 95% confidence interval (95% CI). A lower limit of the 95% CI (ROR05) equal to or greater than 1 was deemed indicative of a positive signal.

$$ROR=\frac{N_{observed}}{N_{expected}}$$

In our study, preferred terms (PTs) of hepatitis adverse events with no fewer than ten cases (N > 10) that both meet the above two criteria (IC025 > 0 and ROR05 > 1) of disproportionality analysis were defined as ICI-related HRAEs.

### 2.4 Descriptive analysis

A comprehensive descriptive analysis was performed to summarize the clinical characteristics of FAERS reports documenting ICI-associated HRAEs. Variables analyzed included gender, country, outcome, FDA receipt date, immunotherapy regimen, report type, and other relevant clinical features. The association between ICI therapies and HRAEs was evaluated using both the IC and the ROR when the full database served as the comparator. However, IC cannot compare reporting between individual drugs (Bate et al., 1998; Norén et al., 2006; Norén et al., 2013). As a result, only the ROR was used when comparing individual drugs or drug classes to each other.

### 2.5 Statistical analysis

Samples with missing data were omitted from statistical analyses for each clinical characteristic. A *p*-value <0.05 was the threshold for statistical significance, with all statistical tests being two-tailed. We performed the statistical analyses and visualizations using R software (version 4.3, ggplot2 package), Microsoft Excel (version 16.65) and GraphPad Prism 9 (version 9.4.1). This study is reported as per the Strengthening the Reporting of Observational Studies in Epidemiology (STROBE) guideline (Von Elm et al., 2007).

## 3 Results

### 3.1 Identification of ICI-related HRAEs in the FAERS database

**FIGURE 2:**
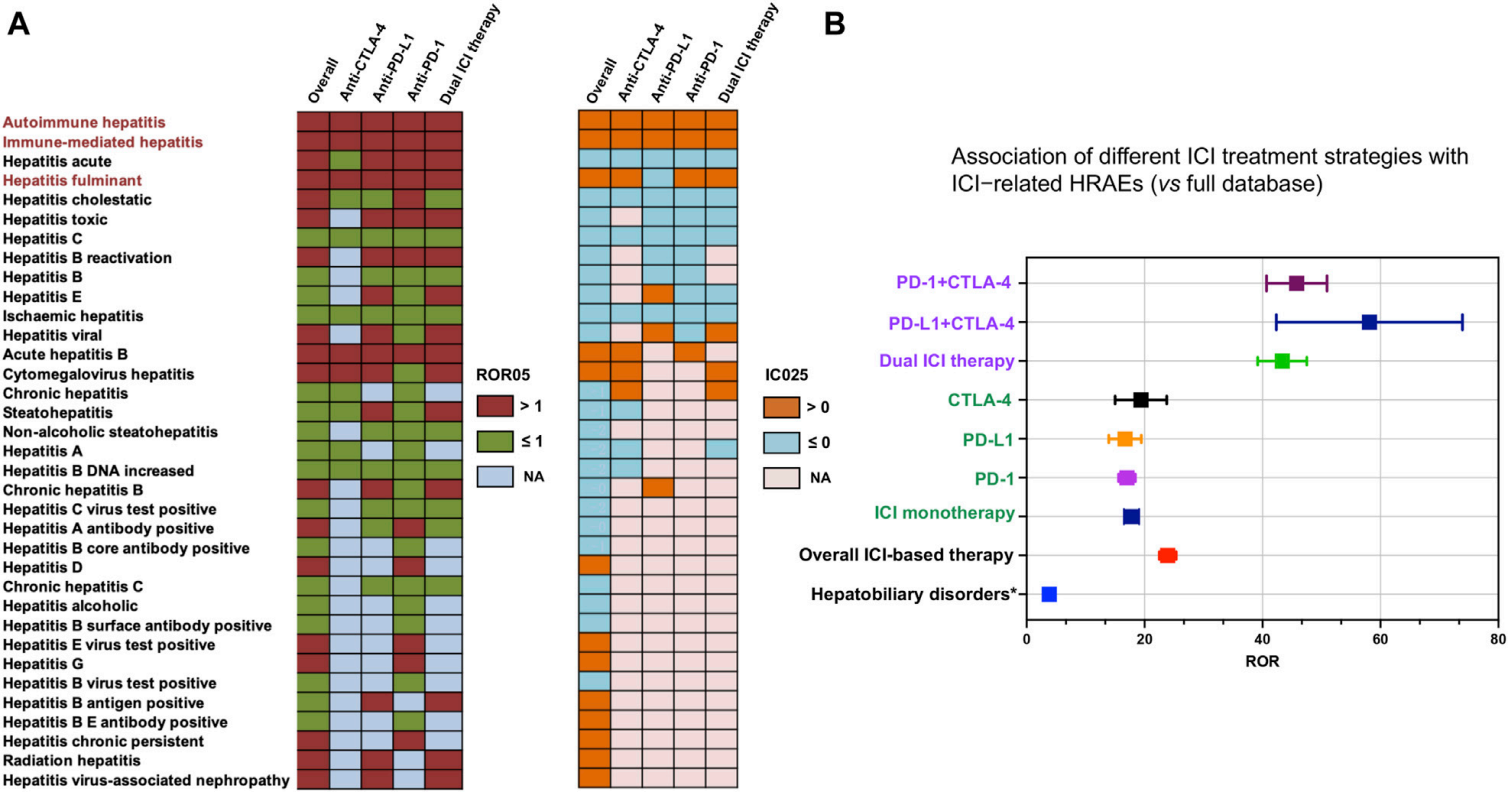
Scanning for ICI-related HRAEs based on the FAERS database. **(A)** The heatmap shows the ROR05 and IC025 for 35 hepatitis adverse events in the FAERS database under different ICI treatment strategies (including overall situation, dual ICI therapy, and ICI monotherapy). Hepatitis-related adverse events were identified and labeled with dark red color to meet the criteria including IC025 > 0, ROR05 > 1, and the number of cases occurring no less than 10. **(B)** The forest plot shows the ROR of hepatitis-related adverse events (considering the three categories of ICI-related HRAEs as one category of adverse events) under different ICI treatment strategies (including overall situation, dual ICI therapy, ICI monotherapy). * The overall hepatobiliary disorders caused by ICIs were used as a reference. HRAEs, hepatitis-related adverse events; ROR05, lower limit of 95% CI of reporting odds ratio. IC025, the lower limit of 95% CI of the information component (IC).

We firstly systematically tabulated the various categories of HRAEs and quantified their prevalence in the reports concerning the use of ICIs. The results revealed that autoimmune hepatitis (N = 634, 38.66%), immune-mediated hepatitis (N = 546, 33.29%), hepatitis acute (N = 85, 5.18%), hepatitis fulminant (N = 80, 4.88%), and hepatitis cholestatic (N = 65, 3.96%) emerged as the top five categories, displaying the highest frequency of reported cases. Subsequently, we conducted a disproportionality analysis, computing the ROR and IC for each PT associated with no fewer than ten cases within HRAEs. The full FAERS database served as the reference dataset for this analysis. Following stringent filtering based on predefined criteria for a positive signal, we identified distinctive HRAEs associated with various ICI treatment strategies, as depicted in Figure 2A. Finally, we designated three PTs (autoimmune hepatitis, immune-mediated hepatitis, and hepatitis fulminant) as ICI-related HRAEs, characterized by a statistically significant increase in reporting after ICI treatment, relative to their occurrence in the full database.

The utilization of ICIs was associated with an increased occurrence of autoimmune hepatitis, immune-mediated hepatitis, and hepatitis fulminant in comparison to their occurrence in the full database (Table 1). Specifically, the ROR05 for autoimmune hepatitis was 17.80, with an associated IC025 of 2.43. Similarly, immune-mediated hepatitis exhibited an ROR05 of 189.95 and an IC025 of 4.75, while hepatitis fulminant displayed an ROR05 of 3.65 and an IC025 of 0.49. Notably, immune-mediated hepatitis emerged as the ICI-related hepatitis adverse event with the most substantial ROR and IC signals across all contexts. Using the complete FAERS database as the reference, we recalculated the ROR and IC signals for ICI-related hepatitis adverse events. In an overarching analysis, all ICI treatment strategies exhibited a statistically significant association with the occurrence of ICI-related HRAEs, revealing an ROR of 23.6 (95% CI 22.3–25.1) (Figure 2B). Further delineating the nuances of ICI treatment strategies, we observed that ICI monotherapy exhibited similar ROR values. In contrast, the dual ICI therapy (combination ICI immunotherapy with anti-PD-(L)1 and anti-CTLA4) was notably associated to the highest ROR among the various treatment regimens, with an ROR of 41.3 (95% CI 37.5–45.4) (Figure 2B).

**TABLE 1:** Hepatitis adverse events reported with ICIs *versus* those reported in the full database from the FAERS, from 2004Q1 to 2023Q1.

	AEs reported for ICIs (n = 353,949)	AEs reported in full database (n = 49,568,379)	IC025	ROR05
Autoimmune hepatitis	634	4,566	2.43	17.80
Immune-mediated hepatitis	546	350	4.75	189.95
Hepatitis acute	85	5,213	−0.50	1.83
Hepatitis fulminant	80	2,439	0.49	3.65
Hepatitis cholestatic	65	4,702	−0.73	1.50
Hepatitis toxic	38	2,210	−0.42	1.73
Hepatitis C	32	11,234	−3.00	0.28
Hepatitis B reactivation	28	2,412	−0.98	1.11
Hepatitis B	19	5,132	−2.62	0.33
Hepatitis E	14	1,192	−0.97	0.96
Ischaemic hepatitis	10	1,055	−1.27	0.71
Hepatitis viral	10	641	−0.56	1.16
Acute hepatitis B	9	232	0.71	2.77
Cytomegalovirus hepatitis	8	300	0.19	1.84
Chronic hepatitis	6	681	−1.38	0.55
Steatohepatitis	6	393	−0.60	0.95
Non-alcoholic steatohepatitis	5	953	−2.12	0.30
Hepatitis A	5	753	−1.78	0.38
Hepatitis B DNA increased	5	676	−1.63	0.43
Chronic hepatitis B	5	218	−0.03	1.31
Hepatitis C virus test positive	4	642	−1.88	0.32
Hepatitis A antibody positive	4	177	−0.05	1.17
Hepatitis B core antibody positive	3	352	−1.43	0.38
Hepatitis D	3	53	1.20	2.46
Chronic hepatitis C	2	337	−1.95	0.21
Hepatitis alcoholic	2	284	−1.70	0.24
Hepatitis B surface antibody positive	2	282	−1.69	0.25
Hepatitis E virus test positive	2	38	1.08	1.77
Hepatitis G	2	5	3.37	10.79
Hepatitis B virus test positive	1	352	−3.01	0.06
Hepatitis B antigen positive	1	40	0.05	0.48
Hepatitis B E antibody positive	1	30	0.43	0.63
Hepatitis chronic persistent	1	15	1.32	1.22
Radiation hepatitis	1	14	1.41	1.31
Hepatitis virus-associated nephropathy	1	8	2.06	2.17

Data are n unless otherwise stated. ICIs, refer to any AEs, reported for treatment with nivolumab, pembrolizumab, atezolizumab, avelumab, durvalumab, ipilimumab, or tremelimumab. The positive IC025 value (>0) and ROR05 (>1) are the traditional thresholds used in statistical signal detection with the FAERS., FAERS, the FDA, adverse event reporting system; ICIs, immune checkpoint inhibitors; IC, information component; ROR, reporting odds ratios; IC025, the lower end of a 95% credibility interval for the IC; ROR05, the lower limit of the 95% confidence interval for ROR.

### 3.2 Descriptive analysis of cases with ICI-related HRAEs

**FIGURE 3:**
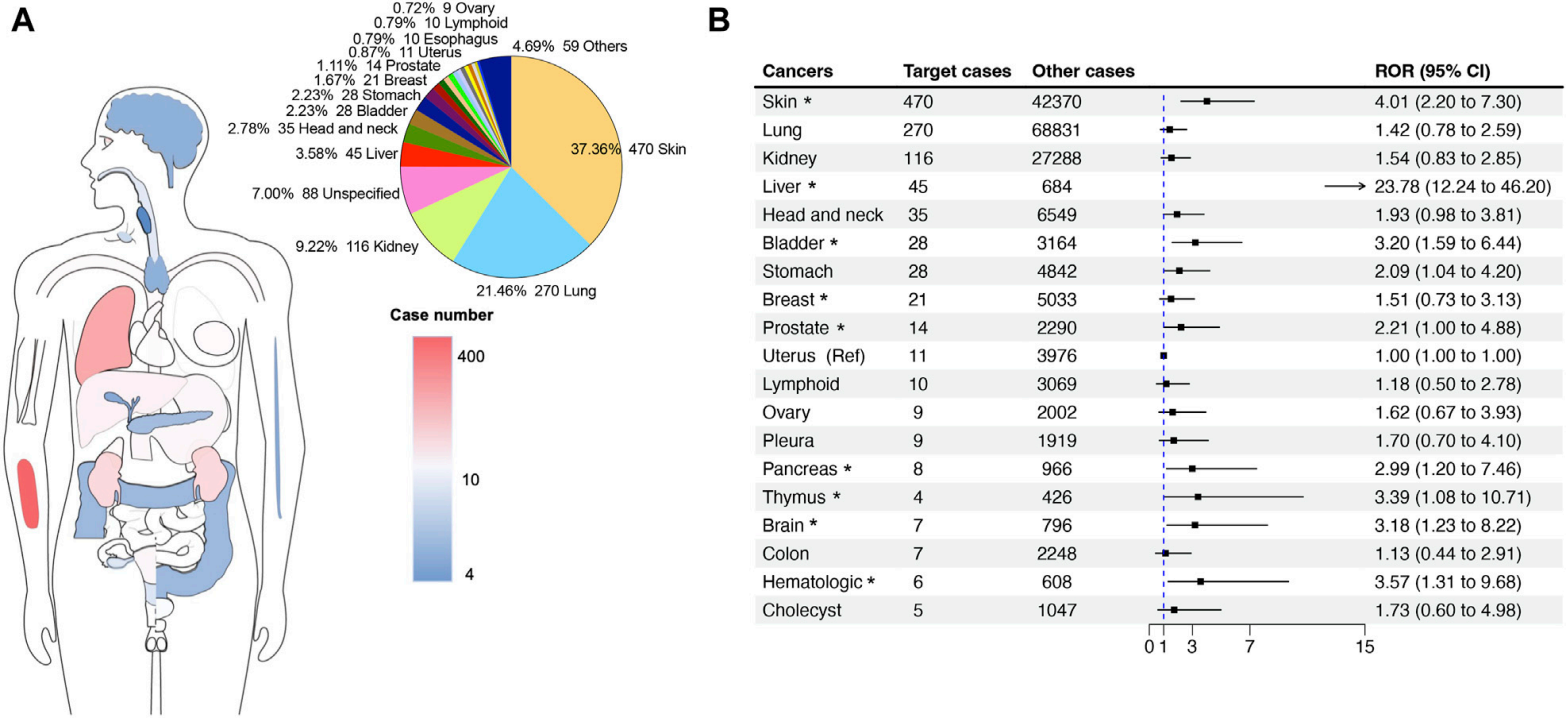
The site statistics for cancer occurrence in reports with hepatitis-related adverse events associated with ICIs. **(A)** The anatomical diagram of the patient’s original cancer site and the number of cases. The pie chart on the right shows the proportional composition of the patient’s cancer original sites. **(B)** The forest plot shows the ROR of ICI-related hepatitis adverse events among different cancer indications. The uterus cancer was used as a reference. * indicates a significant difference in comparison to uterus cancer.

Following a meticulous screening of the FAERS database, we identified a total of 1,260 cases exhibiting HRAEs related to the use of ICIs. Subsequently, we conducted a comprehensive statistical analysis to describe the clinical characteristics, as summarized in Table 2. The median age of the involved patients was 63 years (interquartile range [IQR] 53.8–72) as indicated in 88 available cases. Most of the reported cases were male, constituting 57.2% of the total (N = 719). Furthermore, a significant proportion of these cases originated from the Americas, accounting for 34.6% (N = 435). Notably, the substantial majority of reports, approximately 87.8%, were submitted by healthcare professionals within the last 2 years (40.4%). Of the 1,260 cases, 24.9% (N = 313) experienced a fatal outcome, underscoring the severity of ICI-related HRAEs. A detailed analysis of ICI treatment strategies revealed that the majority of cases involved monotherapy with anti-PD-1 or anti-PD-L1 agents, constituting 51.0% (N = 641) of the total cases. Additionally, the dual ICI therapy was prominent, representing 34.2% (N = 430) of the cases. Among the cases experiencing ICI-related hepatitis adverse events, the indications for treatment predominantly encompassed skin cancer (37.4%, N = 470), followed by lung cancer (21.5%, N = 270), and kidney cancer (9.2%, N = 116) (Figure 3A).

**TABLE 2:** Clinical characteristics of patients with ICI-associated autoimmune hepatitis, immune-mediated hepatitis, or hepatitis fulminant from the FAERS database.

Clinical characteristics	Overall (n = 1,260)	Autoimmune hepatitis (n = 634*)	Immune-mediated hepatitis (n = 546*)	Hepatitis fulminant (n = 80*)
Reporting region				
Americas	435 (34.6%)	223 (35.2%)	206 (37.7%)	6 (7.5%)
Oceania	53 (4.2%)	31 (4.9%)	22 (4.0%)	0 (0.0%)
Africa	4 (0.3%)	3 (0.5%)	1 (0.2%)	0 (0.0%)
Europe	513 (40.8%)	285 (45.0%)	209 (38.3%)	21 (26.3%)
Asia	251 (20.0%)	90 (14.2%)	108 (19.8%)	53 (66.3%)
Missing	2 (0.2%)	2 (0.3%)	0 (0.0%)	0 (0.0%)
Reporters				
Healthcare professional	1,105 (87.8%)	529 (83.4%)	504 (92.3%)	73 (91.3%)
Non-health-care professional	149 (11.8%)	101 (15.9%)	42 (7.7%)	7 (8.8%)
Reporting year				
2011–2015	69 (5.5%)	67 (10.6%)	0 (0.0%)	2 (2.5%)
2016–2020	681 (54.1%)	422 (66.6%)	224 (41.0%)	37 (46.3%)
2021–2023Q1	508 (40.4%)	145 (22.9%)	322 (59.0%)	41 (51.3%)
Sex				
Male	719 (57.2%)	361 (56.9%)	302 (55.3%)	56 (70.0%)
Female	429 (34.1%)	224 (35.3%)	190 (34.8%)	17 (21.3%)
Missing	110 (8.7%)	49 (7.7%)	54 (9.9%)	7 (8.8%)
Age at onset, years	63 (53.75–72); n = 88	64 (55.25–71.5); n = 26	60 (52–72.5); n = 55	69 (60.5–70.5); n = 7
Drugs				
Monotherapy with anti-PD-1 or anti-PD-L1	643 (51.0%)	319 (50.3%)	282 (51.6%)	42 (52.5%)
Nivolumab	247 (19.6%)	139 (21.9%)	87 (15.9%)	21 (26.3%)
Pembrolizumab	268 (21.2%)	121 (19.1%)	134 (24.5%)	13 (16.3%)
Cemiplimab	19 (1.5%)	8 (1.3%)	10 (1.8%)	1 (1.3%)
Atezolizumab	79 (6.2%)	42 (6.6%)	34 (6.2%)	3 (3.8%)
Avelumab	12 (1.0%)	5 (0.8%)	7 (1.3%)	0 (0.0%)
Durvalumab	18 (1.4%)	4 (0.6%)	10 (1.8%)	4 (5.0%)
Monotherapy with anti-CTLA-4	83 (6.6%)	70 (11.0%)	10 (1.8%)	3 (3.8%)
Ipilimumab	76 (6.0%)	65 (10.3%)	8 (1.5%)	3 (3.8%)
Tremelimumab	7 (0.6%)	5 (0.8%)	2 (0.4%)	0 (0.0%)
Dual ICI therapy	430 (34.2%)	209 (33.0%)	199 (36.4%)	22 (27.5%)
Nivolumab plus ipilimumab	399 (31.7%)	197 (31.1%)	181 (33.2%)	21 (26.3%)
Pembrolizumab plus ipilimumab	17 (1.4%)	7 (1.1%)	10 (1.8%)	0 (0.0%)
Tremelimumab plus durvalumab	10 (0.8%)	3 (0.5%)	6 (1.1%)	1 (1.3%)
Atezolizumab plus Ipilimumab	3 (0.2%)	1 (0.2%)	2 (0.4%)	0 (0.0%)
Cemiplimab plus Ipilimumab	1 (0.1%)	1 (0.2%)	0 (0.0%)	0 (0.0%)
ICIs plus chemotherapy	104 (8.3%)	36 (5.7%)	55 (10.1%)	13 (16.3%)
Outcome				
Death	313 (24.9%)	156 (24.6%)	100 (18.3%)	58 (72.5%)
Life-threatening	179 (14.2%)	104 (16.4%)	59 (10.8%)	17 (21.3%)
Hospitalization	758 (60.3%)	405 (63.9%)	302 (55.3%)	53 (66.3%)
Disability	33 (2.6%)	19 (3.0%)	14 (2.6%)	0 (0.0%)
Others	1,089 (86.6%)	530 (83.6%)	489 (89.6%)	71 (88.8%)
Missing	9 (0.7%)	9 (1.4%)	0 (0.0%)	0 (0.0%)
Indication organ				
Skin	470 (37.4%)	277 (43.7%)	176 (32.2%)	18 (22.5%)
Lung	270 (21.5%)	127 (20.0%)	122 (22.3%)	21 (26.3%)
Kidney	116 (9.2%)	54 (8.5%)	51 (9.3%)	11 (13.8%)
Unspecified	88 (7.0%)	53 (8.4%)	32 (5.9%)	3 (3.8%)
Liver	45 (3.6%)	17 (2.7%)	26 (4.8%)	2 (2.5%)
Head and neck	35 (2.8%)	16 (2.5%)	14 (2.6%)	5 (6.3%)
Bladder	28 (2.2%)	12 (1.9%)	16 (2.9%)	0 (0.0%)
Stomach	28 (2.2%)	11 (1.7%)	13 (2.4%)	4 (5.0%)
Breast	21 (1.7%)	8 (1.3%)	13 (2.4%)	0 (0.0%)
Prostate	14 (1.1%)	4 (0.6%)	10 (1.8%)	0 (0.0%)
Uterus	11 (0.9%)	5 (0.8%)	5 (0.9%)	1 (1.3%)
Esophagus	10 (0.8%)	4 (0.6%)	2 (0.4%)	4 (5.0%)
Lymphoid	10 (0.8%)	3 (0.5%)	6 (1.1%)	1 (1.3%)
Ovary	9 (0.7%)	4 (0.6%)	4 (0.7%)	2 (2.5%)
Pleura	7 (0.6%)	2 (0.3%)	2 (0.4%)	3 (3.8%)
Pancreas	8 (0.6%)	5 (0.8%)	3 (0.5%)	0 (0.0%)
Brain	7 (0.6%)	4 (0.6%)	3 (0.5%)	0 (0.0%)
Colon	7 (0.6%)	1 (0.2%)	6 (1.1%)	0 (0.0%)
Hematologic	6 (0.5%)	3 (0.5%)	3 (0.5%)	0 (0.0%)
Cholecyst	5 (0.4%)	0 (0.0%)	5 (0.9%)	0 (0.0%)
Thymoma	4 (0.3%)	1 (0.2%)	3 (0.5%)	0 (0.0%)
Others	59 (4.7%)	23 (3.6%)	31 (5.7%)	5 (6.3%)

Data are n (%), or median (IQR; range); ICI, immune checkpoint inhibitor; FAERS, FDA, Adverse Event Reporting System. * One patient reported both autoimmune and immune-mediated hepatitis, and another patient reported a combination of autoimmune hepatitis and hepatitis fulminant.

We further explored the RORs of ICI-related HRAEs among different cancer indications by a disproportionality analysis. Compared to the uterus cancer as a reference (N = 11 cases, the lowest ROR in the groups), patients with liver cancer, skin cancer, bladder cancer, prostate cancer, stomach cancer, pancreases cancer, thymus cancer, brain cancer, and hematologic cancer have significant higher RORs, in particular liver cancer. When treated with ICI therapies, the patients with liver cancer have 23.78 times higher odds (ROR = 23.78 [12.24–46.20], *p* < 0.0001) of developing hepatitis, followed by patients with skin cancer (ROR = 4.01 [2.20–7.30], *p* < 0.0001).

We also explored whether the different ICI treatment strategies influence the occurrence of ICI-related HRAEs. Table 3 illustrates the associations of ICI-related HRAEs with various treatment regimens, including anti-CTLA-4 therapy, anti-PD-1 or anti-PD-L1 therapy, and dual ICI therapy in comparison to monotherapy. Patients treated with anti-PD-1 or anti-PD-L1 antibodies exhibited a high frequency of immune-mediated hepatitis in comparison to those undergoing anti-CTLA-4 monotherapy, with a ROR of 3.59 (95% CI 1.78–6.18). Moreover, the dual ICI therapy had a higher reporting rate of immune-mediated hepatitis compared to the ICI monotherapy, with an ROR of 2.74 (95% CI 2.30–7.56). In cases of autoimmune hepatitis and fulminant hepatitis, patients receiving dual ICI therapy were overrepresented compared to those on monotherapy, likely due to greater immune system activation. The RORs for autoimmune hepatitis and fulminant hepatitis were 2.46 (95% CI 2.09–7.31) and 1.74 (95% CI 1.06–4.81), respectively. However, no significant difference in reporting was observed between patients treated with anti-PD-1 or anti-PD-L1 monotherapy and those subjected to anti-CTLA-4 regarding these two adverse events (Table 3).

**TABLE 3:** Selected ICI-related hepatitis adverse events reported for ICIs *versus* the full database from the FAERS database, from 2004Q1 to 2023Q1.

	Cases reported with ICIs (N = 353,950)			Cases reported in the full database (N = 49,568,379)	ROR (95% CI) anti-PD-1 anti-PD-L1 vs. anti-CTLA-4 monotherapy	ROR (95% CI) dual ICIs vs. monotherapy	ROR (95% CI) ICIs vs. full database
	Anti-PD-1 or anti-PD-L1 Monotherapy; N = 268,517	Anti-CTLA-4 Monotherapy; N = 22,903	Dual ICI therapy; N = 62,530				
Autoimmune hepatitis	352 (0.13%)	63 (0.27%)	219 (0.35%)	5,200	0.48 (0.36–6.30)	2.46 (2.09–7.31)*	19.34 (17.80–21.02)*
Immune-mediated hepatitis	336 (0.13%)	8 (0.03%)	202 (0.32%)	896	3.59 (1.78–6.18)*	2.74 (2.30–7.56)*	217.24 (189.95–248.45)*
Hepatitis fulminant	54 (0.02%)	4 (0.02%)	22 (0.04%)	2,519	1.15 (0.42–3.31)	1.74 (1.06–4.81)*	4.56 (3.65–5.70)*

Data are n (%) unless otherwise stated. ICIs, refer to any AEs, reported for treatment with nivolumab, pembrolizumab, atezolizumab, avelumab, durvalumab, ipilimumab, or tremelimumab. Anti-PD-1, or anti-PD-L1, monotherapy refers to any AEs, associated with any of the following five drugs only when used alone: nivolumab, pembrolizumab, atezolizumab, avelumab, or durvalumab. Anti-CTLA-4, monotherapy refers to any AEs, associated with ipilimumab or tremelimumab alone. Dual ICI, therapy refers to any AEs, reported with at least one anti-PD-1, or anti-PD-L1, drug combined with an anti-CTLA-4, drug. FAERS, FDA, adverse event reporting system; ICIs, immune checkpoint inhibitors; ROR, reporting odds ratio. * Significant over-reporting within immunotherapy subgroups.

Further analysis of specific subclassification to individual ICI agents, we used ipilimumab, the only one CTLA-4 inhibitor, as the reference for the comparison. Table 4 shows the risk profile of HRAEs for different ICIs compared to the full FAERS database and specifically to ipilimumab. The analysis of ROR values against the full FAERS database shows a heightened risk of HRARs for all ICIs when compared to the overall database. This indicates a notable association of HRAE with these agents. Specifically compared to ipilimumab, nivolumab and pembrolizumab, along with atezolizumab and durvalumab, have a significant lower ROR, suggesting fewer risks of HRAE relative to ipilimumab. The RORs of cemiplimab and avelumab do not significantly deviate from that of ipilimumab, implying a similar risk profile for HRAE with these agents.

**TABLE 4:** The risk profile of hepatitis-related adverse events associated with different ICIs *versus* the FAERS full database.

	HRAE cases	All AE cases of ICIs	ROR (95% CI) vs. Ipilimumab	ROR (95% CI) vs. full database
Cemiplimab	22	9,906	1.54 (0.99–2.39)	38.51 (25.33–58.55)
Nivolumab	576	471,495	0.84 (0.71–1)*	22.55 (20.72–24.54)
Pembrolizumab	320	298,395	0.74 (0.62–0.89)*	19.21 (17.18–21.48)
Atezolizumab	111	104,568	0.73 (0.58–0.93)*	18.57 (15.4–22.39)
Avelumab	13	6,798	1.32 (0.75–2.32)	31.22 (18.11–53.83)
Durvalumab	38	43,278	0.61 (0.43–0.86)*	15.23 (11.07–20.95)
Ipilimumab	179	123,792	Ref (1.0)	25.5 (21.99–29.58)

Notes: FAERS, FDA, adverse event reporting system; ICIs, immune checkpoint inhibitors; ROR, reporting odds ratio; HRAE, hepatitis-related adverse event; AE, adverse event; CI, confidence interval. * Significant difference compared to Ipilimumab.

## 4 Discussion

Although the hepatotoxicity associated with different ICIs has been investigated (Xu et al., 2023), the study focusing more narrowly on HRAEs and the difference among different types of ICI-based therapies remains limited. By employing the full FAERS database as a reference dataset, our study presents the largest and most comprehensive clinical characterization of HRAEs that were highly associated to the treatment of ICIs through a rigorous disproportionality analysis.

ICIs have garnered widespread adoption in the management of various malignancies, including melanoma, lung cancer, renal cell carcinoma, and urothelial cancer (Postow et al., 2015; Shiravand et al., 2022). This adoption has stemmed from early clinical trials demonstrating substantial enhancements in clinical outcomes with ICI treatments. However, the adverse events associated with ICIs, including hepatitis, have emerged as a notably clinically significant complication. (Jiang et al., 2019). The link between hepatitis and liver cancer is well-established, with chronic hepatitis being a major risk factor for the development of hepatocellular carcinoma (HCC), the most prevalent type of liver cancer (Perz et al., 2006; Tu et al., 2017). For example, hepatitis B virus (HBV) acts as a potent liver carcinogen, primarily through mechanisms involving viral integration, chronic inflammation, and immune-mediated cellular damage (Song et al., 2019). While hepatitis was infrequently reported in the initial clinical trials involving ICI therapies, there has been a discernible increase in the number of published case reports and case series documenting hepatitis cases (Berti et al., 2021; Zhao et al., 2022). These case series have illuminated the diverse clinical presentations of hepatitis adverse events. Nevertheless, the comprehensive spectrum of ICI-related HRAEs remains elusive. In this study, we identify autoimmune hepatitis, immune-mediated hepatitis, and hepatitis fulminant as potential considerations for ICI-related HRAEs. These findings provide valuable insights for clinicians engaged in the management of cancer patients undergoing immunotherapies. Moreover, a substantial proportion of the ICI-related HRAE reports sourced from the FAERS database were concentrated within the past 2 years. This trend suggests that the increased reporting of adverse events over time is likely due to the growing use of ICIs, along with the their expanding range of indications.

Importantly, our study offers the most extensive clinical characterization of ICI-related HRAEs based on a comprehensive analysis of all collected cases within the FAERS database. To the best of our knowledge, this dataset of 1,260 patients represents the largest compilation of such cases to date. Our findings underscore the poor outcomes of ICI-associated HRAEs, with a substantial proportion of cases resulting in adverse outcomes. Specifically, 24.9% of cases were reported with fatal outcomes, while 14.2% were reported with life-threatening outcomes, emphasizing the severity of these events. It is also important to note that a higher risk of immune-related hepatitis is reported in real-world settings compared to clinical trials of ICIs as described by Z. Zhang et al (Zhang et al., 2022). The stringent inclusion and exclusion criteria and the shorter exposure and study period in the clinical trials can be attributed to the discrepancy. Additionally, real-world populations include patients being treated in community settings who may not have the same degree of experience or vigilance for irAEs as academic centers participating in trials. Considering these factors, our study suggests that while clinical trials provide valuable insights into the efficacy and safety of anticancer therapies, real-world data is crucial for understanding the full spectrum of drug-related adverse events in the broader patient population.

Furthermore, we identified that ICI-related HRAEs were linked with various ICI treatments and a diverse range of cancer types. The patients with liver cancer receiving ICIs have the highest risk of developing hepatitis (ROR = 23.78 [12.24–46.20] due to several compounding factors. Primarily, the liver, already compromised by cancer, may have reduced functional reserve, making it more susceptible to further damage from the inflammatory and immune-mediated effects of ICIs (Sangro et al., 2020). Additionally, patients with liver cancer often have underlying chronic liver conditions such as cirrhosis or chronic hepatitis, which themselves are risk factors for increased liver inflammation (Shiani et al., 2017). The cumulative effect of a pre-existing hepatic disease, the burden of liver cancer, and the immune-modulating actions of ICIs likely contributes to this heightened risk, making management and monitoring of liver function particularly crucial in this patient group.

Additionally, the dual ICI therapy emerges as a prominent high-risk factor in comparison to monotherapy (ROR = 2.23, Figure 2B) due to the synergistic enhancement of immune activation. By simultaneously blocking two critical immune checkpoints, dual therapy leads to a more profound disinhibition of immune responses (Chu et al., 2023). This dual blockade not only enhances the efficacy against tumors but also increases the likelihood of breaking self-tolerance, leading to higher rates of autoimmune and inflammatory side effects, including hepatitis. These findings align with prior published case series that have similarly reported a heightened frequency of hepatitis incidents associated with the dual ICI therapies (Da et al., 2020; Ramos-Casals et al., 2020). We further reviewed the immune-related hepatitis events reported in the clinical trials (Weber et al., 2009; Robert et al., 2015; Long et al., 2017; Hodi et al., 2018; Yau et al., 2020; Boyer et al., 2021; Aamdal et al., 2022; Wolchok et al., 2022), the relative risk (RR) of dual ICI therapy (6.5%) *versus* monotherapy (3.8%) was 1.71, slightly lower compared to the ROR obtained from the real-world setting. The identification of patients at elevated risk for ICI-associated HRAEs is of paramount importance. Demographic profiles indicate that those most at risk typically include older adults, possibly with a history of liver disease or prior immune-related adverse events. These patients are often treated for cancers like melanoma, lung cancer, or renal cancer, which may inherently place them at a higher risk due to the nature of their treatment regimens. Enhanced monitoring of liver function parameters, including alkaline phosphatase, alanine transaminase, aspartate aminotransferase, and bilirubin, should be incorporated into clinical management of patients with all types of hepatitis. For those identified as having a particularly high risk of developing severe hepatitis, additional preventive and therapeutic measures should indeed be considered, including proactive management strategies, alternative therapeutic options, multidisciplinary team approach, and patient education and involvement. By incorporating these strategies, the goal is to not only monitor but actively prevent and manage ICI-induced hepatitis, thereby reducing the risk of fatal outcomes and improving overall patient safety. These recommendations advocate a more aggressive approach to managing patients at the highest risk, aligning with the severity of potential outcomes outlined in our findings.

Through the subgroup analysis, the data suggests that ICIs have a distinct profile of HRAEs when compared to the broader set of data from the FAERS database. Specifically, nivolumab and pembrolizumab, among others, show a higher risk of HRAEs compared to ipilimumab, which could imply a better safety profile in this aspect. It is probably due to the distinct mechanisms through which these pathways modulate the immune system. Anti-PD-1 and anti-PD-L1 agents act primarily by blocking the PD-1/PD-L1 pathway, a critical immune checkpoint that regulates T cell activity in peripheral tissues, including the liver (Singh et al., 2021). By inhibiting this pathway, these agents prevent PD-1 on T cells from engaging with PD-L1 on tumor cells and normal hepatocytes, which normally helps to maintain immune tolerance and prevent autoimmune responses. This leads to increased activation and proliferation of cytotoxic T cells within the liver, enhancing the likelihood of immune-mediated liver injury. In contrast, anti-CTLA-4 therapies primarily regulate immune responses at the level of initial T cell activation in lymph nodes (Seidel et al., 2018). These findings were consistent with previous studies reporting that the incidence and severity of irAEs caused by CTLA-4 are lower for PD-1/PD-L1 inhibitors (Liu et al., 2021). However, it is crucial to consider the clinical context, including patient selection and the underlying mechanism of action of ICIs, which could influence the incidence and reporting of adverse events. Further investigation into these differences, perhaps through a stratified analysis of patient subgroups or a deeper mechanistic study, might provide more insight into the safety and monitoring strategies for these therapies.

While the precise mechanisms underlying ICI-associated HRAEs remain incompletely understood, it is imperative to recognize the pivotal role of the liver’s unique immunological attributes in its pathogenesis. The liver holds a distinctive position due to its connection to the portal circulation, which serves as the primary conduit for detoxifying blood entering from the intestines and processing a multitude of antigen exposures (Crispe, 2014). As one of the primary mechanisms contributing to liver immunotolerance, hepatic non-parenchymal cells also express PD-L1, as well as CD4^+^ Treg cells expressing CTLA-4 (Makarova-Rusher et al., 2015). It drives synergistically to shield the liver from autoimmune reactions triggered by antigens by suppressing the activity of effector T cells. However, with the administration of ICIs to disrupt these critical regulatory pathways, T cells may become excessively activated, breaching the liver’s immune tolerance. This susceptibility to acute inflammatory responses subsequently precipitates hepatitis (Gudd and Possamai, 2022). Furthermore, the disruption of self-tolerance in the liver activates a variety of immune cells, contributing to the pathophysiological development of immune-mediated hepatitis (Gudd et al., 2021). Given the emergent nature of ICI-induced immune-mediated hepatitis, the cornerstone of treatment involves the prompt initiation of high-dose glucocorticoids (Darnell et al., 2020). Additionally, the consideration of other hepatoprotective agents, including isoglycyrrhizinate, bicyclol, or reduced glutathione, which are commonly used in patients with liver inflammation, may also be considered (Niu et al., 2021).

Our study also has several limitations that warrant consideration: firstly, the FAERS database is a global spontaneous reporting system, open to reports from healthcare professionals, consumers, pharmaceutical companies, and individuals who suspect potential adverse reactions. It introduces inherent selection biases, including variations in the ethnicity and geographical origin of reported cases. Consequently, we are unable to establish a definitive causal relationship between ICIs and ICI-related HRAEs. Moreover, the database does not facilitate the calculation of incidence rates for these identified ICI-related HRAEs, although the incidence of ICI-related HRAEs could be determined as approximately 3%–6% using the data from clinical trials. Secondly, the wide array of anticancer drugs, such as targeted therapy agents, chemotherapeutic drugs, and antibody drugs, presents a challenge in individually extracting all such drugs from the FAERS database. This intricacy can introduce a potential bias related to indications and increase the risk of false positive associations. Thirdly, although the patients with liver cancer were identified with the highest ROR of developing hepatitis when receiving ICI therapies, we cannot eliminate the influence of the disease state that may induce hepatitis. Finally, the three identified ICI-related HRAEs have not undergone clinical validation. The extremely increased ROR observed in FAERS for severe HRAEs might be influenced by the limitations of the FAERS database, particularly reporting biases and the absence of denominator data which would provide a more accurate risk assessment. Further research, including prospective cohort studies, case-control studies, and nested case-control studies, is essential to validate findings from the FAERS database and to accurately determine the risk profile of ICIs in relation to severe hepatitis. These studies would monitor patients from the initiation of ICI therapy, tracking the onset and progression of hepatitis and any subsequent development of liver disease. This approach allows for the collection of baseline liver function data, aiding in controlling for pre-existing liver conditions. Stratifying patients based on their hepatitis status before starting ICI treatment would enable comparisons between those with and without prior hepatitis. Such studies would provide richer patient background information, which is often inadequately captured in the FAERS database.

## 5 Conclusion

While it is recognized that hepatitis induced by ICIs is associated with the risk of liver cancer, significant uncertainties remain concerning the long-term effects and the precise mechanisms involved. Our study contributes to the existing body of evidence by providing a detailed analysis of hepatitis as an adverse event following ICI therapy, using a large, real-world dataset from the FAERS database. This real-world evidence is critical, as clinical trials often have stringent inclusion criteria and may not fully capture the breadth of adverse outcomes seen in a more diverse patient population. To further enhance understanding and management of ICI-induced hepatitis, additional research should focus on longitudinal studies to observe the long-term effects and progression of hepatitis in patients receiving ICIs, identifying precise mechanistic pathways through detailed molecular and cellular studies. Development of predictive biomarkers is also crucial to more effectively identify at-risk patients, thereby facilitating personalized treatment approaches. Additionally, comparative studies across different ICIs and treatment regimens could also provide valuable insights into risk profiles and guide safer treatment protocols. These focused areas of research are essential for developing targeted strategies to reduce the incidence and severity of hepatitis in patients treated with ICIs.

In conclusion, our study provides comprehensive real-world data that illuminate the prevalence and characteristics of ICI-related HRAEs, including autoimmune hepatitis, immune-mediated hepatitis, and hepatitis fulminant, reinforcing the need for heightened surveillance and management strategies in clinical practice. By documenting the variety and severity of hepatitis cases associated with different ICIs and treatment regimens, it adds depth to the clinical understanding necessary for optimizing patient care in oncology. Furthermore, the study underscores the potential for severe outcomes, including death, from ICI-induced hepatitis, which emphasizes the critical need for ongoing research and improved clinical protocols.

## Data Availability

The original contributions presented in the study are included in the article/Supplementary material, further inquiries can be directed to the corresponding author.
